# Do Medical Sciences Students Have the Proper Knowledge, Attitude, and Practice, Regarding the Complementary and Alternative Medicine in Iran?

**DOI:** 10.4314/ejhs.v33i3.17

**Published:** 2023-05

**Authors:** Ali Jadidi, Nazi Nejat, Javad Javidian, Hamed Ahmaditabar, Arash Abbasineyestani, Masoomeh Kalantari, Amirhosein Latifi, Azam Moslemi

**Affiliations:** 1 Department of Nursing, School of Nursing, Arak University of Medical Sciences, Arak, Iran; 2 Medical Science Researcher, Arak University of Medical sciences, Arak, Iran; 3 Traditional and Complementary Medicine Research Center, Arak University of Medical Sciences, Arak, Iran; 4 Department of Biostatistics and Epidemiology, School of Health, Scientific Research Center, Tehran University of Medical Sciences, Tehran, Iran

**Keywords:** Complementary Therapy, Alternative Therapy, University Student, Knowledge, Attitude, practice

## Abstract

**Background:**

The increasing use of complementary and alternative medicine (CAM) has changed expectations of healthcare professionals concerning the knowledge, attitude, and practice of CAM. The present study aimed to investigate the knowledge, attitude, and practice of students of Arak University of Medical Sciences (Iran) in 2020 concerning CAM.

**Methods:**

The present descriptive cross-sectional study was conducted on 226 medical sciences students using targeted quota sampling. The data collection tool was a researcher-made questionnaire based on valid scientific articles and literature. The questionnaire was comprised of 92 items and assessed students' knowledge, attitude, and practice regarding CAM.

**Results:**

The study participants' mean CAM knowledge and attitude scores were 14.12±6.1 and 58.7±29.28, respectively. The primary information sources included mass media, the internet, and friends. Most participants (63.4%) were willing to use CAM methods. The most common CAM was herbal therapy (29.1%), and the most common reason *for* using, was gastrointestinal problems (39.2%).

**Conclusion:**

The participants indicated moderate knowledge and poor attitude regarding CAM. Thus, considering the extensive application of CAM methods among the participants and their insufficient knowledge and inappropriate attitude, that is recommended to include CAM methods be included besides the conventional medicine, in the academic curriculum of students of medical sciences.

## Introduction

Traditional medication methods for preventing and treating various diseases are widespread in Iran. These methods date back more than 3000 years ago, originating from Iranian pre-Islam medicine, Greek medicine, and Egyptian medicine (1). Iranian medicine gradually faded over time with the emergence of modern medicine. This trend started in the 18^th^ century with the foundation of medical and pharmaceutical faculties that followed western methods. Even though Iranian medicine faced legal issues, it persisted as an inseparable part of culture despite the considerable pressure of modern medicine (1,2).

Traditional medicine is a collection of knowledge, skills, and methods based on different cultures' theories, beliefs, and experiences. Traditional medicine supports health and helps prevent, diagnose, recover, or treat physical or mental illnesses. The terms complementary and alternative medicine (CAM) are interchangeable with traditional medicine in some countries; the medicine is part of the countries' tradition and is not the same as standard healthcare (3). 'Complementary Medicine’ is a unique form of medicine used with standard medicine, while ‘Alternative Medicine’ is an uncommon medicine used instead of standard medicine (4).

Increased public and medical attention to CAM is among the most remarkable signs of structural changes in the healthcare system. CAM focuses on the lifestyle, emotional, and spiritual aspects of the disease, which are the most vital elements of CAM (5). The World Health Organization (WHO) has reported increased use of CAM worldwide (6). It is assumed that CAM plays a pioneering role in healthcare and especially self-care in the 21^st^ century (7). In addition, many studies are focused on CAM; thus, recent basic sciences and clinical studies have improved the understanding of these therapeutic methods and their different methods (8).

Fernandez Cervilla et al. (2013) reported the following prevalence of CAM use for disease treatment and maintaining and improving health status: Ethiopia (90%), Chile (71%), Canada (70%), Colombia (40%), China (40%), Germany (33%) (9). Similarly, 66.3% of the participants living in Tehran, Iran had used at least one of the CAM methods and 52.2% of those had used at least one CAM during the last year (10). Another study in this regard in Iran has reported the most positive attitude regarding prayer therapy (14.5%) the most average attitude regarding herbal therapy (73.6%) and the most negative attitude regarding medicinal leech therapy (85.5%) (11).

The utilization of CAM therapies is widespread among the general population, even for specific diseases such as diabetes, respiratory infections, pain, asthma, multiple sclerosis, nosocomial infections, and cancer (12–20). The growing utilization of evidence-based CAM has changed expectations of healthcare professionals regarding the requirement of CAM instructions (21). The American Society of Health-System Pharmacists (ASHP) has suggested that CAM be included in the educational curriculum of the health care providers to properly support patients' safety and health (22).

Various studies indicated that many students had positive attitudes toward CAM (21,24,25) and consistently reported their needs to learn more about CAM (26). Therefore, it is vital to include CAM in the nursing (27), and medical sciences students' educational curriculum although its implementation requires hard work because many university lecturers and professors do not have the necessary knowledge to provide and develop such courses. (28). On the other hand, some studies suggest an inadequate understanding of medical students regarding CAM. For example, Adib et al.'s study in Iran revealed that 88.4% of the participants were unaware of CAM, and 77.8% of them reported their willingness to learn about CAM (24). Barikani et al. also indicated that 96.4% of the physician participants had positive attitudes towards CAM, 11% had good knowledge, 36.3% had moderate knowledge, 52.7% had poor knowledge, and only 17.9% of them had recommended CAM to their patients (25). The finding of a study in South Korea revealed that more than 50% of nursing student had lack of sufficient information regarding CAM, moderate to high negative attitudes regarding CAM, and rarely used CAM (26). Despite a positive attitude toward CAM among medical students, Samara et al. also reported the knowledge gap regarding CAM (27).

Hence, considering the controversial findings on individuals' knowledge, attitude, and practice, particularly students of medical sciences, the present study aimed to investigate the knowledge, attitude, and practice of students of Arak University of Medical Sciences in 2020.

## Methods

The present descriptive cross-sectional study was conducted at the Arak University of Medical Sciences (Iran) in 2020. After obtaining a research permit, the researchers referred to different faculties of Arak University of Medical Sciences, described the research plan, and sampled the students willing to participate in this study. Quota sampling was used based on the study inclusion criteria considering the number of students from different faculties. The data collection tool was a researcher-made questionnaire based on scientific articles and literature. Ten faculty members of Arak University of Medical Sciences approved the visual and content validity of the questionnaire.

The reliability of the questionnaire was assessed by test-retest; the questionnaires were completed twice with two weeks intervals, and the results were compared. The correlation coefficient value was ≥ 0.7, reflecting suitable reliability. The first part of the questionnaire consisted of demographic information (including age, marital status, educational semester, and student's main major). The second part of the questionnaire consist of a set of questions (92 items), about students' knowledge (32 items), attitude (48 items) and practice (12 items) regarding CAM.

**Ethical considerations:** This study is authorized by the Arak University of Medical Sciences Ethics Committee, (IR.ARAKMU.REC.1397.47). Written informed consent was obtained from the participants. Participants were informed about the purpose of the study and their participation was anonymous, voluntarily and confidential.

## Results

Table 1 presents the demographic characteristics of the 226 students who participated in the study.

**Table 1:** Demographic characteristics of study participants

Variables		Mean ± SD
Age		22.89±3.3
		Frequency (%)
Gender	Male	117(51.5)
	Female	109(48.5)
Marital Status	Married	26(11.5)
	Single	200(88.5)
Level of degree	Bachelor's degree	126(45.9)
	Professional degree	100(44.1)
Area of Living	Urban	215(95.1)
	Rural	11(4.9)
	Nursing	52(22.9)
	Doctor of Medicine	74(32.6)
Field of Study	Anesthesia Technology	10(4.4)
	Doctor of Dental Surgery	26(11.5)
	Surgical Technology	20(8.8)
	Laboratory sciences	14(6.2)
	Midwifery	10(4.4)
	Speech Therapy	4(1.8)
	Health Engineering	16(7)

The results revealed that the mean knowledge score of CAM was 14.12±6.1 (ranged 0-26). The primary sources of information regarding CAM were mass media (60.8%), the internet (53.3%), and friends (40.1%). A total of 41 participants reported learning different CAM methods. Moreover, the mean attitude score of CAM was 58.7±29.28 (ranged 0-156).

Most of the participants (63.4%) were willing to use CAM. Moreover, 116 participants (51.1%) reported a history of utilizing these methods, and 97 participants (42.7%) reported using at least one CAM method during the last year. A total of 92 participants (40.5%) reported effective utilization of CAM methods for their health issues. On the other hand, 33 participants (14.5%) reported mild side effects. Fifty-nine participants (26%) recommended using CAM methods to others. The most frequent reasons for the recommendation of CAM methods included gastrointestinal issues (67%), pain (56%), and anxiety (44%). The most frequently used type of CAM was herbal therapy (29.1%), followed by cupping (17.2%) and prayer therapy (11%). (Table 2).

**Table 2:** Frequency of use of CAM in participants

Therapeutic method	Frequency (%)
Cupping	39(17.2)
Medical Leech Therapy	19(8.4)
Phlebotomy	6(2.6)
Massage therapy	30(13.2)
Herbal therapy	66(29.1)
Meditation	15(6.6)
Hypnotism	2(0.9)
Skull therapy	1(0.4)
Traditional bathing	5(2.2)
Acupressure	1(0.4)
Therapeutic touch	4(1.8)
Pilates	12(5.3)
Homeopathy	2(0.9)
Aromatherapy	11(4.8)
Prayer therapy	25(11)
Umbilical therapy	11(4.8)

The participants suggested the lack of scientific evidence (87.2%), inaccessibility of reliable centers (85%), and inaccessibility to reliable providers (81.9%) as the primary obstacles in the development and adoption of CAM. The most common reasons of using a CAM method were gastrointestinal problems (39.2%), followed by pain (25.6%) and Skin disorders (22.5%) (Table 3).

**Table 3:** Frequency of use of CAM for different diagnosis of the participants

Disorder	Frequency (%)
Pain	58(25.6)
Depression	22(9.7)
Anxiety	45(19.8)
Skin disorders	51(22.5)
Gastrointestinal disorders	89(39.2)
Diabetes	5(2.2)
Sleep disorders	33(14.5)
Fatigue	37(16.3)
Dyspnea	6(2.6)
Allergy	24(10.6)
Weight change	37(16.3)
Low appetite	19(8.4)
Hypertension	9(3.9)
Other	11(4.8)

## Discussion

This study aimed to investigate the knowledge, attitude, and practice of the students of Arak University of Medical Sciences in 2020. The results of the study revealed that the participants had an average knowledge score regarding CAM. Moreover, the mean attitude score was estimated to be unacceptable. More than half of the participants reported a history of using CAM and More than 40% of them stated a history of learning various techniques of traditional medicine. In this regard, Şenay Topuz et al.'s study in Turkey (2015) demonstrated that most students were aware of acupuncture and domestic therapies. Moreover, students indicated a positive attitude toward employing CAM methods for patients with cancer (28). Similarly, Alzahrani et al., in their study on 242 students, reported that two-thirds (62.4%) of the participants were aware of acupuncture, and 17.4% knew chiropractic. However, their knowledge of herbal medicine was limited. Older students had a more positive attitude towards CAM compared with younger students. Their willingness to learn CAM was acceptable. A high percentage of the students believed combining CAM and conventional medicine is beneficial. However, CAM should be chosen based on evidence and documents (29). Weber et al.'s study revealed that more than 60% of medical students had a neutral attitude regarding CAM, and only 22% indicated a positive attitude in this regard (30). The inconsistencies may be attributed to the different instructions in this regard.

The present study also indicated that the participants' primary sources of information regarding CAM were mass media, the internet, and friends. Moreover, the least important source was the healthcare system. Thus, the participants obtained their knowledge mostly from unspecialized sources. In contrast, Topuz et al.'s study demonstrated that only 20.9% of the participants obtained information regarding CAM from media and the internet, and more than half of the participants believed this information was insufficient (28). Toss Mass Jong et al.'s study showed that 69% of the participating students acquired information on CAM via their families and friends, 25% in the workplace, and 37% in the healthcare centers, though only 1.7% of them stated that they invited patients to utilize CAM (31).

On the contrary, 66% of the students believed that the healthcare team should be aware of CAM therapies (32). Another study revealed that many participants believed that CAM should be included in their educational curriculum. Moreover, researchers believed that students had insufficient information regarding CAM despite their willingness to learn about CAM (34). Hence, CAM should be included in the educational curriculum of medical students.

Another finding of the present study indicated that more than 40% of the participants reported that CAM methods effectively resolved their health issues. They used various CAM methods to improve their symptoms. The most common methods included herbal therapy, cupping, and massage therapy. They frequently used CAM methods for gastrointestinal irritations, pain, and anxiety. Jong et al.'s study indicated that the most commonly used CAM methods included supplementary diets, massage, probiotics, herbal medicine, and psychosomatic (31).

The participants reported different barriers and obstacles in the development and implementation of CAM, the most important of which included lack of scientific evidence, inaccessibility of reliable centers, and lack of access to reliable providers. In this regard, Poreddi et al.'s study reported the absence of solid evidence regarding CAM, lack of education, and appropriate equipment and devices as CAM implementation limitations. The results of their study suggest that CAM education should be included in the educational curriculum of the students of medical sciences (32–33). Thus, documented plans should be included in the academic curriculum of the students of medical sciences to improve their knowledge and attitude. In addition to instructing them on appropriate implementation to provide required education and cares to patients and their families.

The results of the study indicated that most participants had used CAM methods for mild health issues and recommended them to others. However, they had moderate knowledge and poor attitude, possibly due to poor and inadequate education in this field. Thus, considering the extensive application of CAM methods among the participants and their insufficient knowledge and inappropriate attitude, it is recommended be included CAM methods besides the standard medicine, in the educational curriculum of medical sciences students.

The present descriptive study was conducted with small sample size, and thus, may not be generalized to all medical sciences students. Part of the data for this study was collected during the coronavirus epidemic, which may have affected the knowledge, attitude, and performance of students about complementary and alternative medicine.
